# Choosing Wisely: five recommendations related to tests, treatments, and procedures at risk of inappropriateness in the cure of Parkinson’s disease (LIMPE-DISMOV Academy)

**DOI:** 10.1007/s10072-019-03760-3

**Published:** 2019-02-14

**Authors:** Laura Avanzino, Pietro Cortelli, on behalf of the Board of the LIMPE-DISMOV Academy

**Affiliations:** 1 Ospedale Policlinico San Martino-IRCCS, Genoa, Italy; 2 Section of Human Physiology, Department of Experimental Medicine, University of Genoa, Genoa, Italy; 3 IRCCS Istituto Delle Scienze Neurologiche di Bologna, Bologna, Italy; 4 Department of Biomedical and Neuromotor Sciences, University of Bologna, Bologna, Italy

Dear Editor,

We are reporting five recommendations related to tests, treatments, and procedures at risk of inappropriateness in the cure of Parkinson’s disease. These recommendations were identified by the Academy for the Study of Parkinson’s Disease and Movement Disorders (LIMPE-DISMOV Academy), in collaboration with Slow Medicine. Slow Medicine, an Italian movement of health professionals, patients, and citizens promoting a “Measured, Respectful and Equitable Medicine”, launched the campaign “Doing more does not mean doing better- Choosing Wisely Italy” in Italy at the end of 2012, similar to Choosing Wisely in the USA. The campaign aims to help physicians, other health professionals, patients, and citizens engage in conversations about tests or procedures commonly used in their field whose necessity should be questioned and discussed. This call to action has resulted in specialty-specific lists of “Things Providers and Patients Should Question.”

The five recommendations related to Parkinson’s disease were selected during a meeting of the board of the LIMPE-DISMOV Academy on the basis of a list drawn up by the individual members of the board. Each member of the board has indicated a practice, commonly carried out in Italy, for which there are well-founded reasons to consider possible its inappropriate use, which does not bring significant benefits to patients, but rather a greater incidence of side effects or inadequate care. In the selection process, the board of the LIMPE-DISMOV Academy took into careful consideration the level of evidence of the practices that was recently revised in the “Linea Guida Diagnosi e Terapia della Malattia di Parkinson” published in 2013 and updated in 2015, drafted by LIMPE in collaboration with the Istituto Superiore di Sanità.

1. Do not use the brain single photon emission tomography with tracers for dopaminergic transporters (DAT-SPECT) for the prognosis and to ascertain the progression of Parkinson’s disease.

The DaTscan, a dopamine transporter (DAT) single photon emission computerized tomography (SPECT) imaging technique, proved to be valid in the differential diagnosis between Parkinson’s disease and selected neurological conditions (essential tremor, dystonic tremor, and psychogenic parkinsonism). However, several studies have shown that there is insufficient evidence to support the use of DaTscan as a prognostic factor or as a measure of disease progression in Parkinson’s disease [1].

2. Do not use antipsychotic medication except clozapine and quetiapine to treat psychosis in Parkinson’s disease.

Several studies have shown that clozapine and quetiapine, when used to treat psychosis in Parkinson’s disease do not worsen motor symptoms, unlike other antipsychotics. In patients with psychosis and Parkinson’s disease, treatment with low-dose clozapine should be considered as the first choice in the treatment of psychosis. However, a mandatory requirement for clozapine use is regular monitoring of white blood cell count and absolute neutrophil count. If regular weekly blood tests are not possible, low-dose quetiapine should be considered as an alternative antipsychotic therapy for the treatment of psychosis in Parkinson’s disease [2].

3. Do not delay prescribing Levodopa therapy to treat patients early in the course of Parkinson’s disease.

Prescription of levodopa as a pharmacological treatment for Parkinson’s disease is often delayed in favor of levodopa-sparing therapies (such as dopamine agonists) due to concerns regarding the risk of drug-induced motor complications or possible toxic effect of levodopa. However, the increase in the risk of motor complications associated to levodopa therapy compared to dopamine agonist therapy is still debated, while several studies showed that the use of dopamine agonists increases the incidence of other important side effects (such as impulse control disorders) and induces scarcer control of motor symptoms compared to levodopa. Furthermore, clinical studies have not brought conclusive evidence on the risk of neurotoxicity by early treatment with levodopa. Patients in the early stage of Parkinson’s disease may be considered for treatment with levodopa if required by the clinical condition. Noteworthy, the combined treatment of levodopa and entacapone in the early stage of the disease is not indicated, in order to reduce the risk of motor complications [3].

4. Do not use myocardial scintigraphy with metaiodobenzylguanidine (MIBG) to diagnose Parkinson’s disease.

[123I]-MIBG myocardial scintigraphy was originally developed to assess postganglionic presynaptic cardiac sympathetic nerve endings in heart disease. Subsequently, cardiac MIBG uptake was demonstrated to be reduced in patients with Lewy body diseases such as Parkinson’s disease. However, several studies have shown that the sensitivity of MIBG myocardial scintigraphy is comparable to that of clinical diagnosis. Therefore, cardiac [123I]-MIBG myocardial scintigraphy may assist in the differential diagnosis of Parkinson’s disease versus other parkinsonisms, but it must not be used not to replace clinical diagnosis, especially in the early stages of the disease or in the case of diagnostic uncertainty. Furthermore, particular attention should be paid to the pharmacological treatments of patients, in particular tricyclic antidepressants, which can interfere with the result of the [123I]-MIBG myocardial scintigraphy [4].

5. Do not use anticholinergic drugs to treat the motor symptoms of drug-induced parkinsonisms.

Although anticholinergic drugs have been widely used in the control of parkinsonian symptoms in Parkinson’s disease and in parkinsonisms (including drug-induced parkinsonism), the current evidence shows that such drugs have a limited benefit on tremor and are associated with cognitive and neuropsychiatric side effects.

Thus, the use of anticholinergics should be limited in patients with comorbidities as cognitive impairment or clinically significant psychiatric illness. Furthermore, the use of anticholinergic drugs is not recommended for the treatment of motor symptoms in drug-induced parkinsonisms [5].

## Conclusions

The mission of Choosing Wisely is to promote conversations between clinicians and patients by helping patients to choose care that is supported by evidence, not duplicative of other tests or procedures already received, free from harm and truly necessary. The LIMPE-DISMOV Academy as national society with the mission of fighting against Parkinson’s disease and of promoting strong interactions between patients and clinicians decided to join with enthusiasm the Choosing Wisely campaign in the hope that the recognition of evidence-based recommendations regarding low-value services in our specialty reduce unnecessary care, avoid harm, and decrease waste.

Future studies will verify if targeting these low-value services and implementing Choosing Wisely recommendations related to tests, treatments, and procedures at risk of inappropriateness in the cure of Parkinson’s disease will achieve the expected positive results.
